# On the Influence of the Cotton Manufactories on the Health

**Published:** 1818-06

**Authors:** J. Jackson


*For the London Medical and Physical Journal.*

### *On the Influence of the Cotton Manufactories on the Health;* by Mr. J. Jackson.

THE extensive introduction of cotton manufactories into this part of the county requiring extensive population for carrying into effect the various operations on that article, when the legislature is solicited to determine the hours of actual labour, the attention of the medical practitioner is called for, to compare the health of those so employed, with that of the other labouring classes.

Without enquiring into the health of those employed in cotton factories, we should be apt to pronounce them a healthy class of people; but, when we find few attain their fiftieth year, or that do not become superannuated at that age, we infer, and rightly too, that there is something preys on the man thus to induce premature old age.

If a subject be brought up, from the age of eight years, to working thirteen hours daily in a factory, he will generally be of small stature; or if, which seldom happens, he should become tall, his bones and muscles will want that substance and strength which constitute a robust man. Before he arrives at the age of thirty years, he will be uniformly of a pale and sallow aspect, with a general unhealthy appearance; if we examine more accurately, we shall find him complain of deep-seated pain in the hypochondria, with a dry sympathetic cough, indigestion, and yellowness of the skin; all which symptoms strongly indicate a deranged state of the liver and spleen. Children appear not to be affected more in proportion than adults; and all diseases to which they are most liable evidently originate from a disordered state of the chylopoiëtic viscera. A diseased state of the mesenteric glands, impeding the passage of chyle into the circulation,—thereby inducing an unhealthy appearance, and a general retardation of growth,—is amongst the most common affections; as are also sudden faintings, accompanied by slight convulsion: these are undoubtedly excited by the combined action of heat and confined air, loaded with effluvia, from their recovering without medicine, on being withdrawn for the benefit of their health.

The heat necessary for the easy working of cotton machinery averages about 78° Farh.; the operation of which, in producing bilious disorders and obstructions of the viscera, is universally acknowledged,—and derangement in the functions of the viscera produce the chronic diseases to which they are subject. A spinner died a few days ago, aged 49, after having been tapped for ascitis three times within the last three months. He was not examined *post mortem*, but the primary affection was undoubtedly a disease of the liver, bidding defiance to the power of medicine.

The secreting surfaces must naturally be affected from the heat and exertion required, eliciting constant perspiration. The sudden daily transitions (noon and night,) from the degree of heat above mentioned to cool air, in winter, perhaps, below the freezing point, may be considered as a powerfully exciting cause in producing acute diseases,—but I am not satisfied that these people are more than ordinarily subject to acute inflammatory diseases.

Last summer, (1817,) when the thermometer stood at 81° in the shade, an extensive factory here was 93°; the consequence was, cholera morbus made its appearance, and pretty severely: out of six cases, three were dangerous within fourteen hours, and one was eventually fatal: the treatment was diluents and opium. These were unquestionably produced by the excessive heat; and more individuals would certainly have been attacked, had not the heat been, by advice, lowered several degrees,—after which, no more cases occurred. It is, in fact, difficult to say which part of the system suffers most in one employed in a factory; for it is the whole constitution which appears affected, and may be attributed to heat, and confined air, to which I may add, long and constant exertion.

In addition to the above, females are subject more than usually to varicose veins on the legs, which often cause troublesome ulcers, and spasm or cramp of the gastrocnemeus, and soleus muscles. They are also liable to leucorrhœal and disagreeable discharges, with laxity of the vagina; some of the discharges are so acrid, (rendered so, perhaps, for want of cleanliness), as to produce sores on the penis after connection: those sores are imagined by the patient to be venereal, but the history of the case, or appearance of the sore, point out the nature of the disease, which always heals by observance of cleanliness and astringent lotions.

Scrofulous and phthisical complaints are not more common than amongst other mechanics: the former disease, however, is exceedingly prevalent among the weavers of cotton, which may be accounted for by their working in damp cellars, and, lately, may very justly be added, scanty and unhealthy vegetable food.

I shall conclude by giving a statement of the relief received from the Sick-Societies by those employed in cotton-factories, compared with the relief received by other mechanics and artists, taken collectively, which will tend to prove that there is near triple the sickness in the former, to what there is in the latter.

From the aggregate of ten extensive cotton-factories, the workmen, who are members of Sick-Societies, have received, upon an average, the relief to the amount of 11s. 6d. each, per annum: other mechanics, &c. to the amount of 4s. each, per annum.

*Bolton-le-Moors, Lancashire;*

*April* 10, 1818.

---

*For the London Medical and Physical Journal.*

